# Perturbing the metabolic dynamics of *myo*-inositol in developing *Brassica napus* seeds through *in vivo* methylation impacts its utilization as phytate precursor and affects downstream metabolic pathways

**DOI:** 10.1186/1471-2229-13-84

**Published:** 2013-05-21

**Authors:** Jinzhuo Dong, Wei Yan, Cheryl Bock, Kateryna Nokhrina, Wilf Keller, Fawzy Georges

**Affiliations:** 1 Wilmar International, 56 Neil Rd, Singapore 088830, Singapore; 2 National Research Council Canada, Plant Biotechnology Institute, 110 Gymnasium Place, Saskatoon, SK S7N 0W9, Canada; 3 Ag-West Bio Inc, 101 – 111 Research Drive, Saskatoon, SK S7N 3R2, Canada

**Keywords:** *Brassica napus*, Seed coat, Protein translation, Ononitol, Seed development, *myo-*Inositol methyltransferase, Phytic acid, Sucrose, Raffinose oligosaccharides, Galactinol

## Abstract

**Background:**

*myo*-Inositol (Ins) metabolism during early stages of seed development plays an important role in determining the distributional relationships of some seed storage components such as the antinutritional factors, sucrose galactosides (also known as raffinose oligosaccharides) and phytic acid (PhA) (*myo*-inositol 1,2,3,4,5,6-hexa*kis*phosphate). The former is a group of oligosaccharides, which plays a role in desiccation at seed maturation. They are not easily digested by monogastric animals, hence their flatulence-causing properties. Phytic acid is highly negatively charged, which chelates positive ions of essential minerals and decreases their bioavailability. It is also a major cause of phosphate-related water pollution. Our aim was to investigate the influence of competitive diversion of Ins as common substrate on the biosynthesis of phytate and sucrose galactosides.

**Results:**

We have studied the initial metabolic patterns of Ins in developing seeds of *Brassica napus* and determined that early stages of seed development are marked by rapid deployment of Ins into a variety of pathways, dominated by interconversion of polar (Ins phosphates) and non-polar (phospholipids) species. In a time course experiment at early stages of seed development, we show Ins to be a highly significant constituent of the endosperm and seed coat, but with no phytate biosynthesis occurring in either tissue. Phytate accumulation appears to be confined mainly within the embryo throughout seed development and maturation. In our approach, the gene for *myo*-inositol methyltransferase (*IMT*), isolated from *Mesembryanthemum crystallinum* (ice plant), was transferred to *B. napus* under the control of the seed-specific promoters, napin and phaseolin. Introduction of this new metabolic step during seed development prompted Ins conversion to the corresponding monomethyl ether, ononitol, and affected phytate accumulation. We were able to produce homozygous transgenic lines with 19% - 35% average phytate reduction. Additionally, changes in the raffinose content and related sugars occurred along with enhanced sucrose levels. Germination rates, viability and other seed parameters were unaffected by the *IMT* transgene over-expression.

**Conclusions:**

Competitive methylation of Ins during seed development reduces seed antinutritional components and enhances its nutritional characteristics while maintaining adequate phosphate reserves. Such approach should potentially raise the canola market value and likely, that of other crops.

## Background

*myo*-Inositol is an essential component in the biosynthesis of an array of derivatives ranging from simple inositol phosphates to complex membrane-associated products with important cellular functions. It can be isomerized and (or) methylated to form a variety of species-specific epimers and methyl ethers. A number of these, including Ins, have been noted to accumulate mostly in osmotically challenged plants and have since been recognized as osmoprotectant metabolites [1].

Ins is also central to the biosynthesis of a number of antinutritional components such as sucrose galactosides (e.g. RFO) and inositol polyphosphates such as PhA (also known as InsP_6_). The synthesis of PhA predominates in developing seeds, and constitutes the major storage form of seed phosphorus. Both Ins and its bound phosphates are released by hydrolysis upon germination.

The antinutritional properties of PhA reside in its strong binding affinity for positively charged species such as essential minerals (e.g. iron and zinc) and proteins, significantly lowering their bioavailability to humans and animals. As a consequence, the presence of high levels of PhA in canola seed hinders the full exploitation of the pure meal and underrates its potential as a major crop worldwide.

In contrast, PhA has been accredited as an effective antioxidant with antitumor properties and risk reduction of certain types of cancer [2]. Besides decreasing uncontrolled cellular proliferation, PhA is also thought to cause differentiation of malignant cells resulting in reversion to the normal phenotype [3]. Further, PhA has been shown to play a critical role in many cellular events such as signaling [4], apoptosis [5,6], neuroprotection [7], as well as functioning as enzyme cofactor [8]. The antioxidant properties of PhA have also been shown to inhibit free radical formation and lower lipid peroxidation, making it a very efficient natural food and feed preservative [9]. Moreover, PhA has been shown recently to protect developing seeds against oxidative stress [10]. Because of the seemingly paradoxical and unique roles of PhA, and in view of the agronomic value placed on low phytate-containing seeds and (or) meals, total elimination of PhA was not our intended goal.

Phytic acid biosynthesis constitutes a uniquely complex process, consisting of a primary substrate, Ins, and a number of interjecting secondary Ins polyphosphate substrates from other sources and pathways [11,12]. In the primary pathway, the *de novo* synthesis of Ins involves the oxidative cyclization of glucose 6-phosphate (G-6-P) to L*-myo*-inositol-1-phosphate (L-Ins-1-P) by the action of a single enzyme, L*-myo*-inositol-1-phosphate synthase (MIPS). It has been shown that by affecting the production of this enzyme directly or indirectly through mutagenesis in maize [13,14] and soybean [15] or by genetic engineering methods in rice [16,17], soybean [18] and canola [19] PhA accumulation can be reduced by 20–94.5% with a concomitant increase in inorganic phosphate (P_i_). Of the various transgenic approaches reported, *MIPS-*RNAi [18] and *MIPS-*cosuppression [19] transgenics yielded the lowest levels of PhA in the corresponding mature seeds (94.5 and 44%, respectively). However, the RNAi approach reportedly has resulted in hindered seed development [18]. Thus, in view of the fact that Ins is a key substrate in the biosynthesis of many essential cell components, we decided to assess the consequences of its metabolic diversion versus its complete elimination through *MIPS* down-regulation, and compare the effects associated with each approach. We reasoned that while metabolic diversion of Ins may not interfere with its biosynthesis, it could preferably limit its participation in PhA production.

**Scheme 1:**
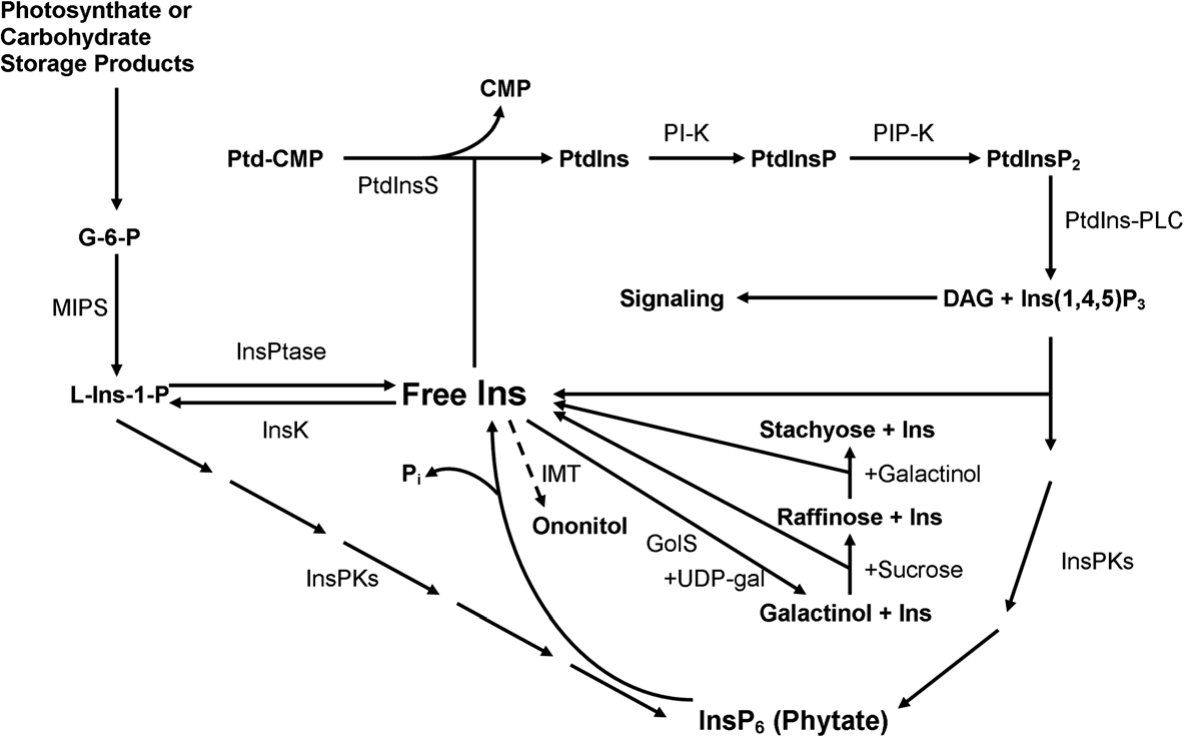
**Metabolic interconversions of phosphorylated *myo*-inositol and related derivatives in developing *B. napus *seeds.** Dashed arrow indicates the newly introduced methylation step. Solid lines represent established metabolic pathways. PI-K, phosphatidylinositol kinase; GolS, galactinol synthase; MIPS, myo-inositol phosphate synthase; InsPKs, *myo*-inositol kinases; PtdInsS, PtdIns synthase; PtdIns-PLC, PtdIns-specific phospholipase C; InsPtase, *myo*-inositol phosphate phosphatase; InsK, *myo*-inositol kinase; G-6-P, glucose-6-phosphate; DAG, diacylglycerol; UDP-gal, uridine diphosphate galactose; Ptd-CMP, phosphatidylcytosine monophosphate; PtdIns, phosphatidylinositol; PtdInsP, phosphatidylinositol monophosphate; P_i_, inorganic phosphate.

The first MIPS product, L-Ins-1-P, appears to be in quasi equilibrium with free Ins due to the activity of a two-enzyme system, in which L*-myo-*inositol-1-phosphate monophosphatase converts L-Ins-1-P to free Ins, while *myo*-inositol-1-kinase regenerates it (Scheme 1). Depending on the rigidity of the requirements for free Ins *vs.* L-Ins-1-P in the developing seed, the relative abundance of each substrate is ultimately determined by the differences in the kinetics of the two opposing reactions. We hypothesized that continual exclusion of free Ins from this quasi equilibrium by metabolic diversion through methylation, could drive the quasi equilibrium in the direction of free Ins. This would subsequently limit the participation of both substrates (Ins and L-Ins-1-P) in PhA biosynthesis. Towards this end, we studied the effect of over-expressing the gene encoding IMT (EC 2.1.1.129) from *M. crystallinum*[20] on seed PhA accumulation in transgenic *B. napus* under two different seed-specific promoters, napin and phaseolin [21]. We also examined the effect of enhancing the translational context of *IMT* on its gene product accumulation and PhA reduction during seed development.

Although the *M. crystallinum IMT* (*McIMT*) has been used to study the osmoprotective properties of methylated cyclitols in transgenic plants [22-25], the current investigation is the first example of seed-specific McIMT-mediated metabolic diversion to reduce phytic acid biosynthesis in seed crops through *in vivo* methylation of Ins.

## Results

### ^3^H-*myo*-inositol metabolism in developing seeds of *B. napus*

**Figure 1:**
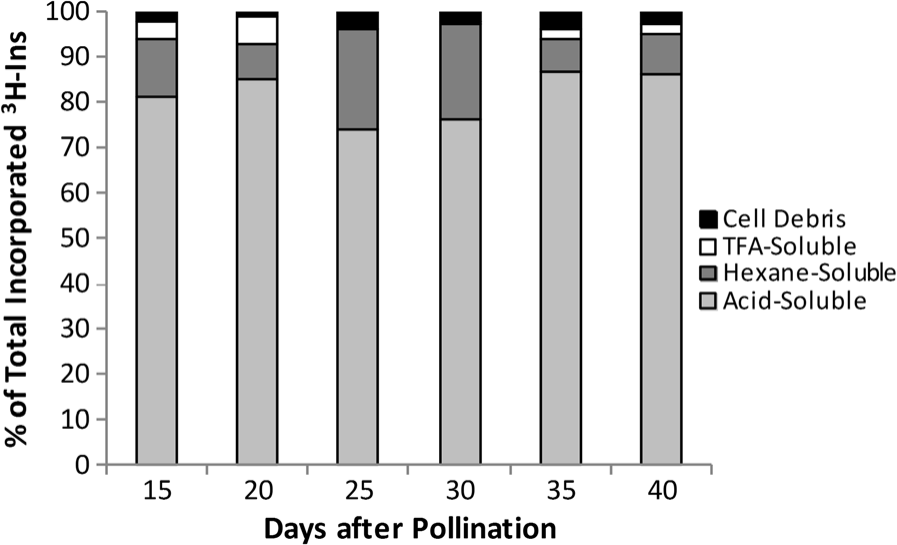
**Differential extraction of **^**3**^**H-*myo*-inositol incorporated in developing seeds.** Developing seeds of *B. napus* were labeled with ^3^H-myo-inositol (^3^H-Ins) and different fractions were extracted as described in Methods. Data represent the percentage of total incorporated ^3^H-Ins in cell debris (black); TFA, (trifluoroacetic acid) (white); hexane (dark grey) and hydrochloric acid (light grey).

*In vivo* labeling of developing *B. napus* seeds with ^3^H-*myo*-inositol, and subsequent fractionation of different cell components (acid-soluble, hexane-soluble, trifluoroacetic acid [TFA]-soluble) and cell debris revealed the relative incorporation of ^3^H-*myo*-inositol in each fraction (Figure 1). The acid-soluble fraction contains free Ins, Ins monophosphates and Ins polyphosphates. The hexane-soluble fraction consists mainly of Ins-containing phospholipids. The TFA-soluble fraction and cell debris mainly include tightly bound membrane components such as glycosyl-phosphatidyl inositol (GPI) protein anchors [26]. Between 15 and 20 DAP, most of the label was recovered in the acid soluble fraction, which contains PhA. After 20 DAP, a decrease in the relative content of the acid-soluble ^3^H-*myo*-inositol-labeled fraction occurred until at least 30 DAP. A simultaneous increase in the relative amount of label incorporation appeared in the corresponding hexane-soluble fraction. After 30 DAP, the relative rates of incorporation in the acid-soluble fraction increased again and remained high until at least 40 DAP.

### Phytic acid accumulation in developing seeds of *Brassica napus*

**Figure 2:**
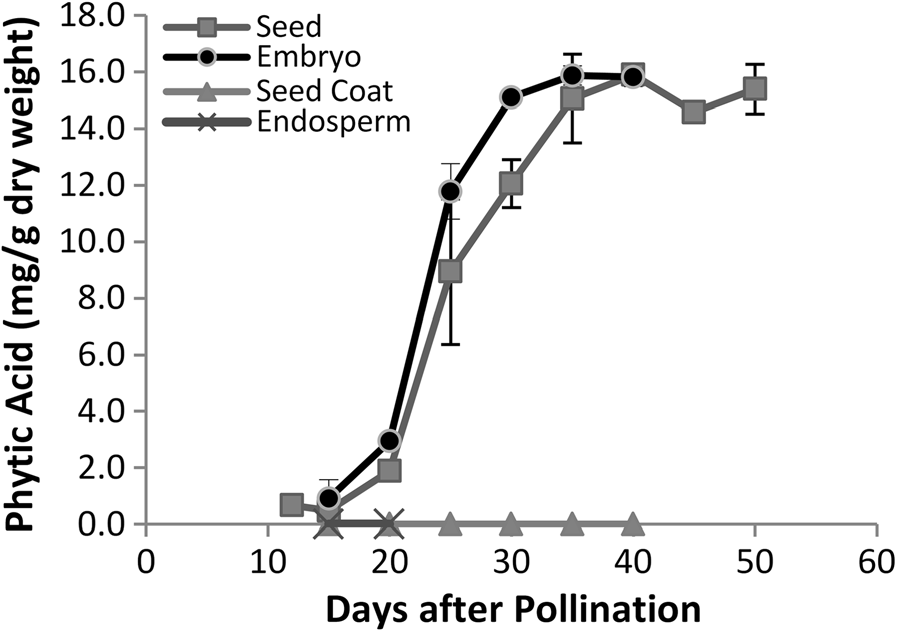
**Phytic acid accumulation during *B. napus *seed development in whole and dissected seed.** The level of phytic acid (PhA) was measured by HPLC in Westar whole seed, seed coat, endosperm and embryo at different developmental stages from12 to 50 days after pollination (DAP). Each data point represents mean value of three biological replicates ±SE (standard error).

**Figure 3:**
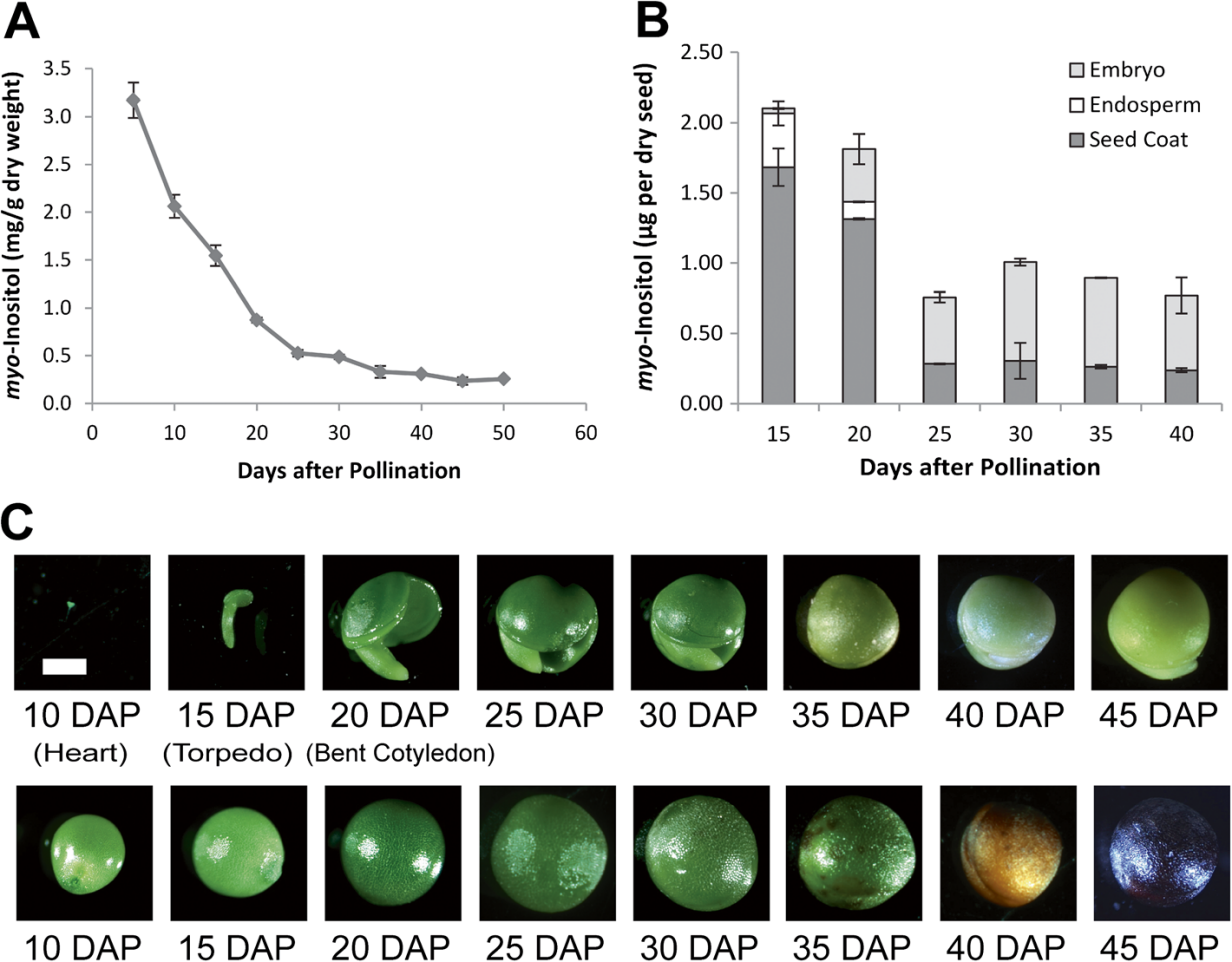
**Analysis of *myo-*Inositol in whole and dissected seed at different developmental stages.** (**A**) Declining levels of *myo-*Inositol (Ins) during *B. napus* seed development expressed as Ins content of whole seed. Ins was analyzed in Westar seed at different developmental stages from 5 to 50 days after pollination (DAP). Each data point represents mean value of three biological replicates ±SE (standard error). (**B**) *myo*-Inositol distribution in different tissues of developing seed. Ins was measured in seed coat, endosperm and embryo at different developing stages. Data represent the amount of Ins distributed in seed coat (dark grey column), endosperm (white column) and embryo (light grey column) of one single seed. Each data point represents mean value of three biological replicates ±SE (standard error). (**C**) Relative sizes of the developing embryo (top row) relative to seed coat (bottom row) at various stages of seed development. The white bar represents the length of 1 mm.

HPLC analysis profiles indicate that PhA started to accumulate in detectable amounts during very early stages of seed development (12 DAP). Its accumulation became more pronounced at 20 DAP and continued progressively, reaching maximum levels at about 35 DAP (Figure 2). This time window (12–35 DAP) is, therefore, important in temporal targeting of molecular strategies for phytic acid reduction in canola seeds. In spite of the fact that Ins was shown to be present in both endosperm and seed coat at all stages examined (Figure 3B), no phytate accumulation was found in either tissue (Figure 2).

### Variations in *myo*-inositol levels during seed development

At early stages of seed development, a steady decline in Ins levels occurred from 5 DAP up to 25 DAP (Figure 3A). These levels continued to decline further at 30 DAP, reaching their lowest point at 45 DAP through maturity. The sharp decline in the levels of Ins during the period 5–20 DAP coincided with the initial gradual accumulation of PhA (Figure 2) as well as the increase in the levels of the non-polar derivatives (Figure 1 at 15–30 DAP). At 30 DAP the rapid decline in Ins levels resumed with a concomitant rise in PhA levels (Figure 2) at the expense of the non-polar components (Figure 1).

Meanwhile, as the embryo continued to expand through the different stages (Figure 3C), the level of Ins declined from its highest point in seed coat and endosperm and gradually increased in embryo tissues starting at 15–20 DAP (Figure 3B). At this point the rate of PhA biosynthesis in the embryo began to increase (Figure 2). No PhA synthesis was observed in either seed coat or endosperm.

### Generation of transgenic lines of *B. napus* carrying the *myo*-inositol methyltransferase gene

Transgenic lines were generated from constructs pN*IMT* (IMT under napin promoter) and pPh*IMT* (IMT under phaseolin promoter). In both napin and phaseolin groups of transgenics, 80% of the lines showed reduced levels of PhA. Three transgenic lines with the highest PhA reduction were chosen from each promoter group for subsequent experiments. These were selfed to homozygosity and were shown to be consistently stable in terms of the *IMT* gene integrity and phytate reduction. One line from each group was chosen for further studies, namely N-11 (napin) and Ph1-18 (phaseolin). Lines Ph2-15 and Ph3-19 originated from two more transformation events and were chosen in the same way. These lines differed from Ph1-18 in that they harbored changes in the translational context of the *IMT* gene (Table 1). In all transgenic lines germination rate was 100% for fresh seed, which did not differ from Westar controls. Additionally, over several generations the *IMT* transgenics did not exhibit changes in seed yield.

**Table 1:** **Modified translational contexts for *IMT *gene driven by the phaseolin promoter**

**Name of construct**	**Line**	**Sequence**
pPhIMT1 (parent-transgenic *IMT*)	Ph1-18	A^-3^A^-2^A^-1^**ATG** A
pPhIMT2	Ph2-15	G^-3^C^-2^C^-1^**ATG** A
pPhIMT3	Ph3-19	A^-3^C^-2^C^-1^**ATG** A

Sequence column shows DNA triplets 5' upstream from the translation initiation codon: line Ph1-18 (parent *IMT*) with dA nucleotides in positions -1 to -3; line Ph2-15 with dC nucleotides in positions -1 and -2 and dG nucleotide in position -3; line Ph3-19 with dC nucleotides in positions -1 and -2 and dA nucleotide in position -3.

### ***IMT*** expression occurs progressively in developing seeds of transgenic *B. napus*

**Figure 4:**
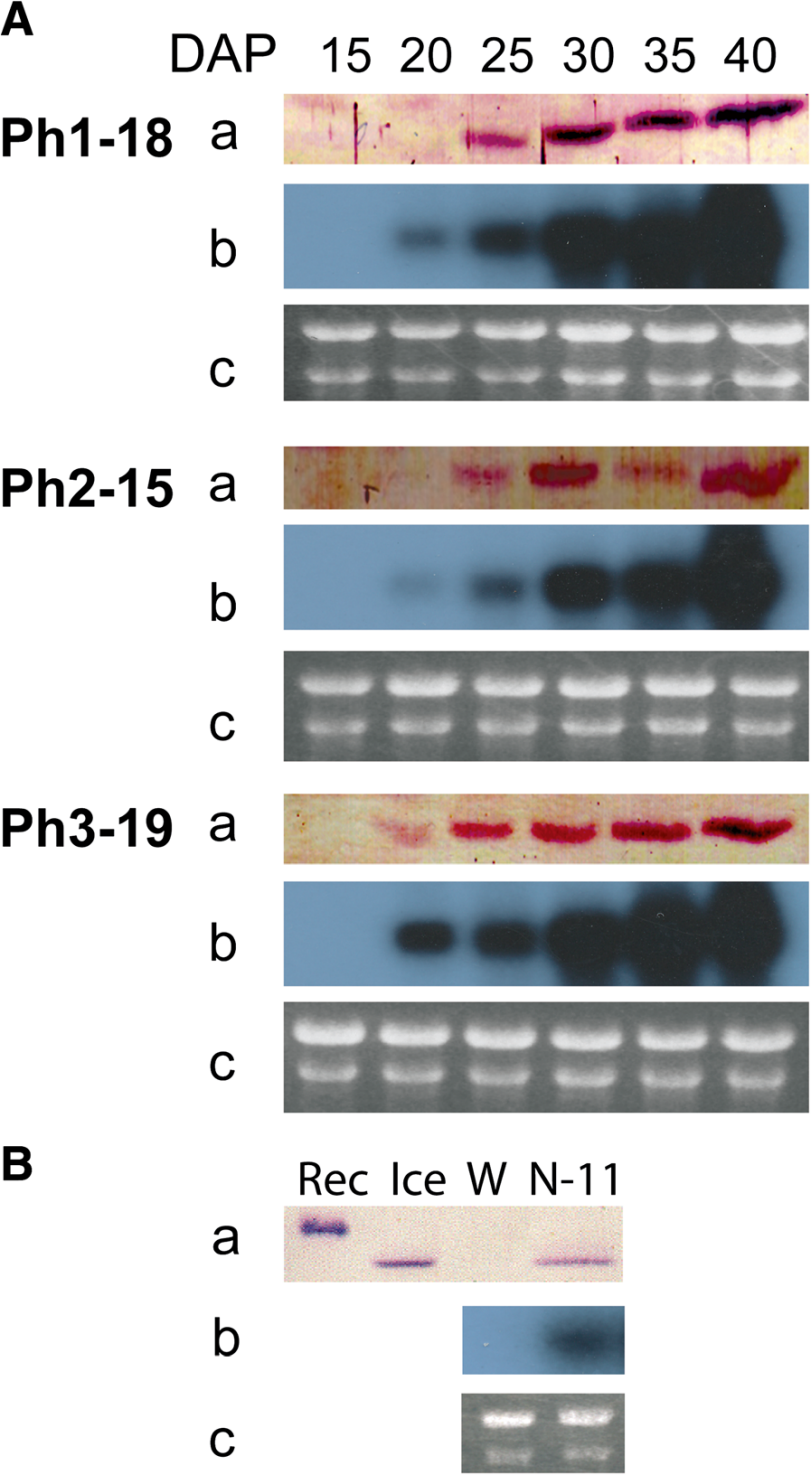
**Gene expression analysis of napin-*IMT *and phaseolin-*IMT *transgenic lines.** (**A**) Gene expression analysis of developing seeds of phaseolin-*IMT* transgenic lines Ph1-18, Ph2-15 and Ph3-19 at 15, 20, 25, 30, 35 and 40 days after pollination (DAP). (**B**) Gene expression analysis of midrange seed development (25 DAP) of the napin-*IMT* transgenic *B. napus* line, N-11. Panels a-c, Western analysis, Northern analysis and RNA gel. Rec, Histidine tagged recombinant IMT produced in *E. coli*; Ice, ice plant protein extract; W, Protein extract from Westar.

In both lines of transformants (using napin and phaseolin promoters) production of IMT was verified by Western blot analysis, which also revealed absence of any native equivalents of IMT in developing seeds of non-transformed *B. napus* (Figure 4A and 4B). Although the *IMT* transcript started to appear at 20 DAP in developing transgenic seeds as exemplified by the phaseolin lines (Figure 4A), Western-blot analysis showed the corresponding protein to be produced in detectable amounts only after 25 DAP. The accumulation pattern of both the *IMT* transcript and its corresponding protein were similar in that they increased progressively to 40 DAP. Likewise, the napin-*IMT* line exhibited a similar manner of protein expression at 25 DAP (Figure 4B).

### Transgenic seeds produce enzymatically active IMT and D-ononitol

**Figure 5:**
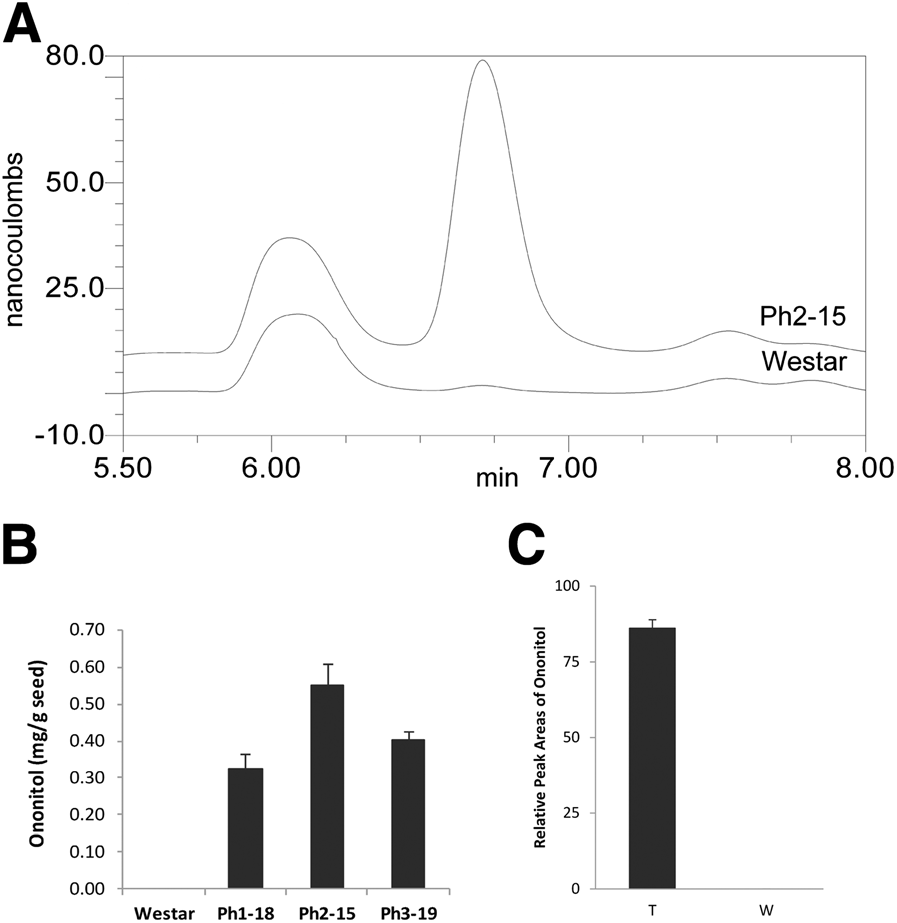
**HPLC evidence of ononitol production by the *IMT *transgene in transgenic lines.** (**A**) Partial trace of mature seed sugar analysis from a representative transgenic line. The peak at 6.75 minutes corresponds to ononitol; (**B**) quantitative levels of ononitol in mature seeds of different phaseolin-*IMT* transgenic lines (Ph1-18, Ph2-15 and Ph3-19). (**C**) IMT enzyme assay of *IMT* transgenic lines (T) vs. wild-type control (W). Relative peak areas represent the average ±SE (standard error) of three biological replicates.

HPLC analysis of mature transgenic seeds revealed the presence and accumulation of a new compound, which co-eluted with authentic D-ononitol standard (Figure 5A and 5B). The newly introduced IMT activity in transgenic lines was further confirmed by the ability of total soluble protein extracts from 40-DAP transgenic seeds to convert Ins to D-ononitol, in an *in vitro* IMT enzymatic assay (Figure 5C). Similar extracts from wild-type seeds as well as from transgenic leaves failed to produce this product. In addition, an unidentified compound eluted immediately prior to the Ins peak in the transgenic mature seed samples (not shown), which was absent in the wild-type samples. This compound did not co-elute with an authentic sample of pinitol.

### Phytic acid content is reduced and inorganic phosphate content is enhanced in the seeds of transgenic plants

**Figure 6:**
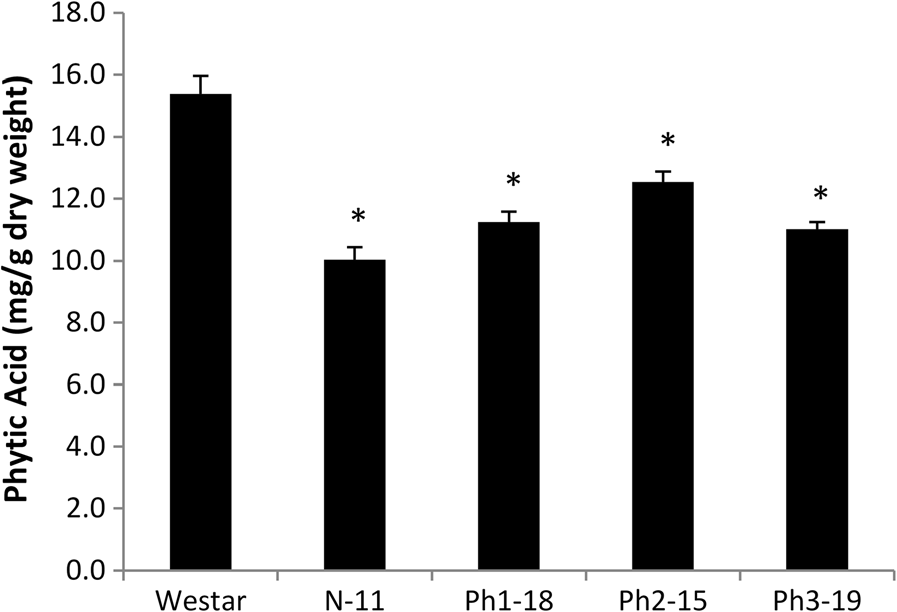
**Phytate level in mature seeds of napin-*IMT *and phaseolin-*IMT *transgenic lines.** Phytate (PhA) was extracted from mature seeds of napin-*IMT* (N-11) and phaseolin-*IMT* (Ph1-18, Ph2-15 and Ph3-19) transgenic lines and measured by HPLC. Each data point represents mean value of five biological replicates ±SE (standard error). Statistical significance was evaluated with the unpaired Student *T*-test (*P<0.05 vs Westar).

**Figure 7:**
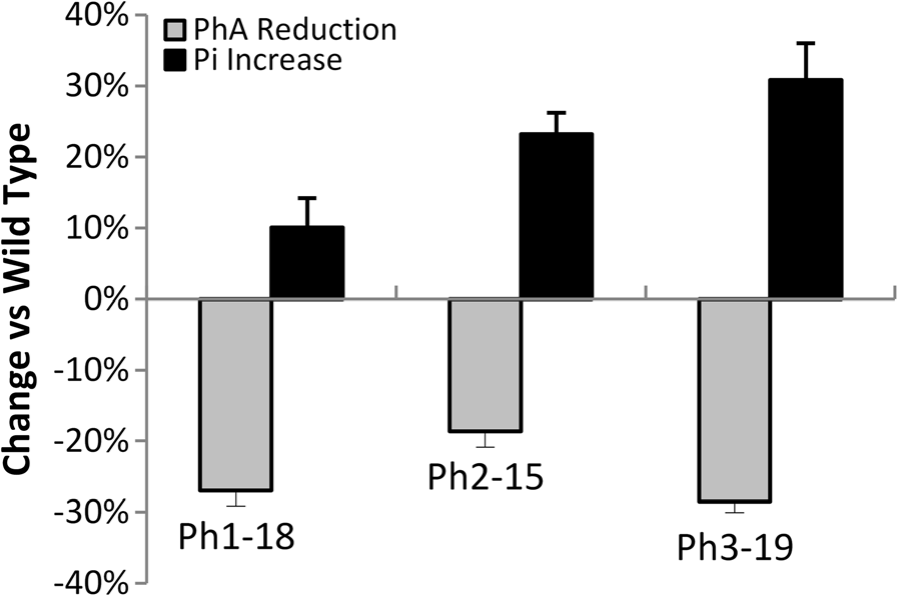
**Changes in phytate and free phosphate in phaseolin-*IMT *transgenic lines.** Columns are expressed as percentage of reduction in phytate (PhA) (grey column) and increase in free phosphate (P_i_) (black column), ±SE (standard error) in phaseolin-*IMT* transgenic lines, Ph1-18, Ph2-15 and Ph3-19.

HPLC analysis for phytate content in mature napin-*IMT* transgenic seeds showed that a 35% reduction in PhA level was achieved. HPLC analysis also showed a reduction of 19-29% in PhA in mature phaseolin-*IMT* seeds despite the translational context modification (Figure 6). Additionally, P_i_ levels increased from 10 to 31% which is consistent with earlier observations (Figure 7).

### Carbohydrate analysis

**Figure 8:**
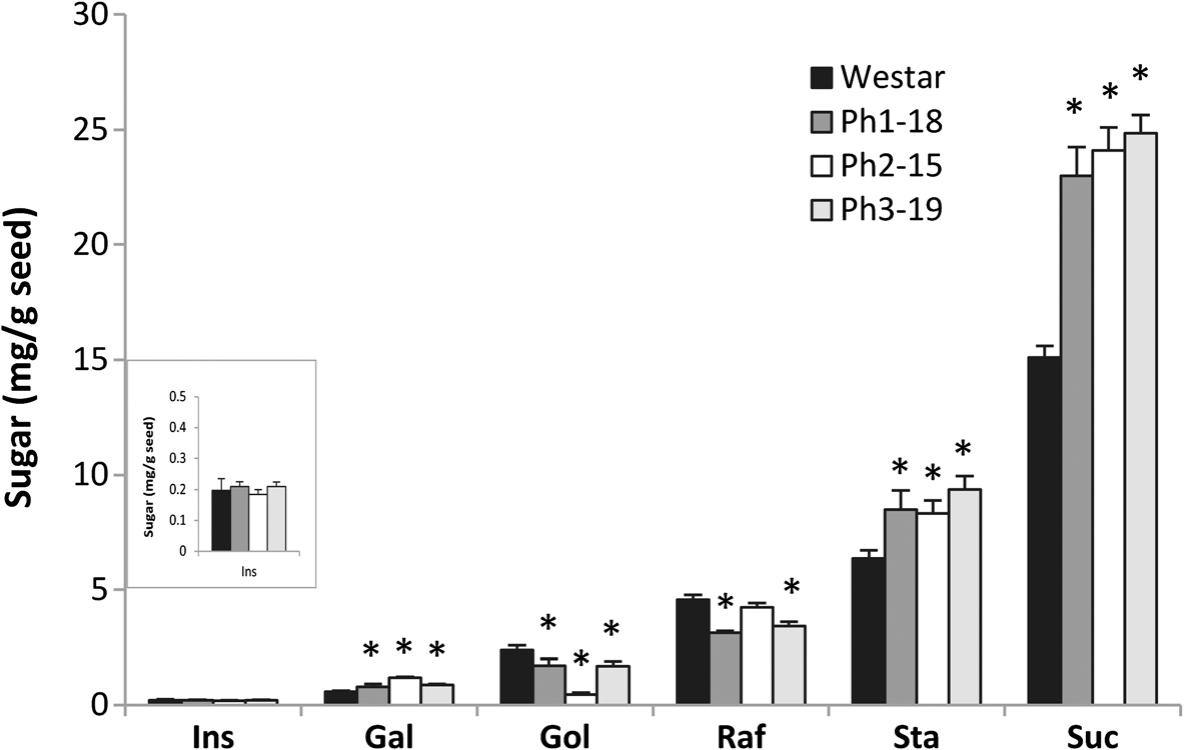
**Sugar levels in mature seeds of phaseolin-*IMT *transgenic lines.** Sugars were extracted from defatted mature seeds from wild-type Westar (black column) and phaseolin-*IMT* transgenic lines, Ph1-18 (dark grey), Ph2-15 (white) and Ph3-19 (light grey) and measured by HPLC. Each data point represents mean value of five biological replicates ±SE (standard error). Statistical significance was evaluated with the unpaired Student *T*-test (*P<0.05 vs Westar). Ins, *myo*-inositol; Gal, galactose; Gol, Galactinol; Raf, Raffinose; Sta, Stachyose; Suc, Sucrose.

Changes in the carbohydrate species associated with Ins metabolism (e.g. galactinol, RFO and sucrose) were observed in mature transgenic seeds. In the three lines examined, the changes were consistent with the competition for available Ins among three pathways (PhA, galactinol and ononitol production, Scheme 1). Such competition appears to have resulted in the reduction of galactinol biosynthesis with consequential downstream effects, which are reflected by increases in galactose and sucrose levels with a concomitant decrease in raffinose level. However, the decrease in raffinose level appears to be accompanied by a proportionate rise in stachyose (Figure 8).

## Discussion

The relative incorporation of ^3^H-Ins in the different fractions of developing *B. napus* seeds, presented in Figure 1, depicts an image of the dynamics of Ins participation in phospholipid and PhA biosynthesis at early to mid stages of development. The data suggests the occurrence of horizontal interconversions between ^3^H-Ins and its polar and non-polar phosphorylated variants. Evidence of this is seen at 20 and 30 DAP where substantial shifts seem to occur across the hexane- and acid-soluble fractions.

The observed decrease in the relative contents of the acid-soluble fraction in the period 20–25 DAP reflects the utilization of *myo*-inositol in the biosynthesis of non-polar compounds (e.g. phospholipids). This is supported by the observation that a parallel increase in the relative amounts of label incorporation appeared in the hexane-soluble fraction during the same period. After 30 DAP, the relative rates of incorporation in the acid-soluble fraction increased again at the expense of the non-polar fraction, reflecting the rapid accumulation of PhA during this period (Figure 2). This is likely, since hydrolysis of Ins-containing phospholipids is known to lead to increased phytate accumulation in seeds of *B. napus *[11].

Typically, the newly forming seed coat is made up of four layers of cells, the outermost epidermal and palisade cell layers (which develop from the outer integument of the ovule) and parenchyma and endothelial cell layers (which are derived from the inner ovule integument) [27]. At 5 DAP the majority of seed inner space is filled with differentiating integument and a small amount of endosperm. That the majority of Ins at 15–20 DAP is localized in the seed coat of dissected seeds (Figure 3B) and that the bulk of the seed at 5–15 DAP is primarily made up of this tissue may be construed as evidence that the seed coat is the primary site of Ins biosynthesis in the *B. napus* seed. In support of this is the abundant supply of sucrose, which is transported through the phloem, apoplastic region and the seed coat during this early stage [28,29] and which, through hydrolysis, acts as source of G-6-P, the precursor for Ins.

As the embryo expands through its initial globular, heart, torpedo and bent cotyledon stages, the endosperm proportionally shrinks (Figure 3C), and Ins becomes localized mainly in the embryo and seed coat (Figure 3B). That the seed coat is the tissue where Ins is most abundant at the early stages of seed development is of special importance since it plays a major role in the biosynthesis of mucilage, other seed coat polysaccharides and sugar acids through its participation in the oxidative pathway [30]. PhA synthesis started at very early stages (<10DAP) and reached its maximum levels at about 35 DAP (Figure 2). Distribution analysis of PhA accumulation between cotyledons and embryo axes revealed that from 20 to 75 DAP more than 80% of PhA is accumulated in cotyledons (80% at 20–25 DAP, increasing up to 90% after 30 DAP) (data not shown). At 25–30 DAP, as the *Brassica* embryo expanded, higher accumulation of the non-polar derivatives (needed for membrane biogenesis) occurred (Figure 1). This period (25–30 DAP) appears to be marked by a slower decline in Ins levels, nearly reaching a plateau (Figure 3A), while, in parallel, PhA levels continued to rise at a relatively slower rate. After 25 DAP and through Ins appears to have reached a stable level in embryo, and more so in seed coat which could account for its apparent slower decline between 25 and 30 DAP (Figure 3B). At this stage the decline in Ins levels was accompanied by a concomitant rise in PhA levels (Figure 2) at the expense of the non-polar components (Figure 1) as the seed approached desiccation through the onset of RFO biosynthesis (30-35DAP) (unpublished results).

Since the initial phosphorylation steps of free Ins commence either with the reconstitution of L-Ins-1-P or through other positional phosphate esters, our strategy was to investigate the effect of competitive metabolic shunt of Ins on its phosphorylation and subsequent PhA and RFO accumulation in canola seeds. To accomplish this, we have explored the conversion of Ins into ononitol (1-D-4-*O*-methyl-*myo*-inositol), by methylation at the D-4 position, through the action of the IMT enzyme.

The observed variations in PhA reduction levels do not necessarily reflect different *IMT* expression levels since all selected lines displayed almost uniform levels of the IMT protein at the time of sampling as shown by Western blot analysis (Figure 4A and 4B). That the recorded ranges of PhA reduction appear to be similar with either promoter (Figure 6) suggests one of two possible scenarios: a) A certain threshold may exist at which a steady balance between supply and removal of Ins is reached, which confines PhA reduction levels within the observed limits; b) The temporal appearance of IMT activity under these promoters may not be in synchrony with the highest point of Ins accumulation, which is presumably reached in less than 10 DAP (Figure 3A), leading to the early onset of PhA synthesis. The latter scenario is likely, since the IMT protein was not detected until 25 DAP in lines Ph1-18 and Ph2-15 (Figure 4A) in contrast to PhA accumulation which is shown to be in progress at 15–20 DAP (Figure 2). A similar result was obtained when a *MIPS* antisense transcript was expressed in transgenic rice driven by the glutelin *GluB-1* promoter [17].

The phaseolin promoter was chosen based on its reported early transcriptional activation in transgenic systems such as tobacco (15–16 DAP) [31,32]. The napin promoter which is native to *Brassica* was chosen with a view to comparing the effect of temporal expression differences of the two promoters on PhA accumulation. However, because *IMT* transcription from the phaseolin promoter did not commence as early as in the case of transgenic tobacco, it was not possible to assess such differences. The complex architecture of the phaseolin promoter has been shown to play a major role in spatial regulation of this promoter in transgenic systems [33]. Further, seed-specific transcriptional regulatory regions in the same promoter have been identified which affect its activation in a temporally dependent manner [34,35]. Therefore, it is possible that the phaseolin promoter, when expressed in different systems, could be affected by elements that may impose temporal expression variations. This is suggested by the fact that, in our hands, the transcriptional activation of phaseolin-*IMT* was triggered in canola at a later time point (20 DAP, Figure 4A) than in tobacco, indicating that this promoter could be influenced by developmentally regulated programs in a host-specific manner.

Attempts at enhancing the *IMT* translation efficiency through the modification of its translational context [36,37] resulted in somewhat improved translation levels at 20 DAP under the phaseolin promoter when a dA nucleotide was positioned at the -3 position and a dC nucleotide at each of positions -2 and -1 of the initiation codon (transgenic line Ph3-19, Table 1 and Figure 4A). Translation levels at subsequent stages in the same line also appear to have been relatively enhanced. However, in spite of the protein level enrichment at 20 DAP in line Ph3-19, this did not substantially improve the overall PhA reduction, confirming the need for a more temporally and (or) spatially synchronized *IMT* expression and Ins production, as opposed to early enhancement of *IMT* translation. Nevertheless, while the apparent compartmentalization of Ins in the seed coat may shield it from IMT action in the embryo, seed-specific promoters are known to drive gene expression in the inner layer of seed coats and there is active transport of Ins from seed coat to embryo [38].

In mutant lines of other crops (e.g. maize [13,39] and soy bean [15]), the decrease in PhA phosphorous in mature seeds is generally accompanied by a parallel, albeit variable, increase in P_i _[15]. However, in some mutant lines (e.g. maize *lpa*2-1), the rise in P_i_ can be accompanied by an accumulation of other Ins phosphates (Ins(1,2,4,5,6)P_5_; Ins(1,4,5,6)P_4_; and Ins(1,2,6)P_3_) [39]. This makes the decrease in PhA futile in such mutants since these highly phosphorylated Ins species retain many of the adverse PhA properties. In the present study, although we observed a similar inverse relationship between PhA and P_i_, there was no detectable accumulation of any of the partially phosphorylated Ins intermediates such as InsP_3_, _4__or 5_. Furthermore, over-expression of *IMT* did not affect the seed viability or the germination efficiency of transgenic canola seeds. In addition, there were no measurable yield penalties (Table 2).

**Table 2:** **Seed yield of *IMT *transgenic lines**

**Line**	**Seed yield (g/plant)**
Westar	10.56 ± 0.76
N-11	11.20 ± 0.80
Ph1-18	10.11 ± 0.62
Ph2-15	9.74 ± 0.69
Ph3-19	9.76 ± 0.69

Mature seeds of Westar, napin-*IMT* (N-11) and phaseolin-*IMT* (Ph1-18, Ph2-15 and Ph3-19) transgenic lines were harvested and weighed. Each data point represents mean value of five biological replicates ±SE (Standard error).

Absence of ononitol in wild-type HPLC chromatograms and failure of the wild-type protein extracts to produce ononitol, together with the Western analysis results suggest possible absence of native IMT-like activity in *B. napus*.

**Figure 9:**
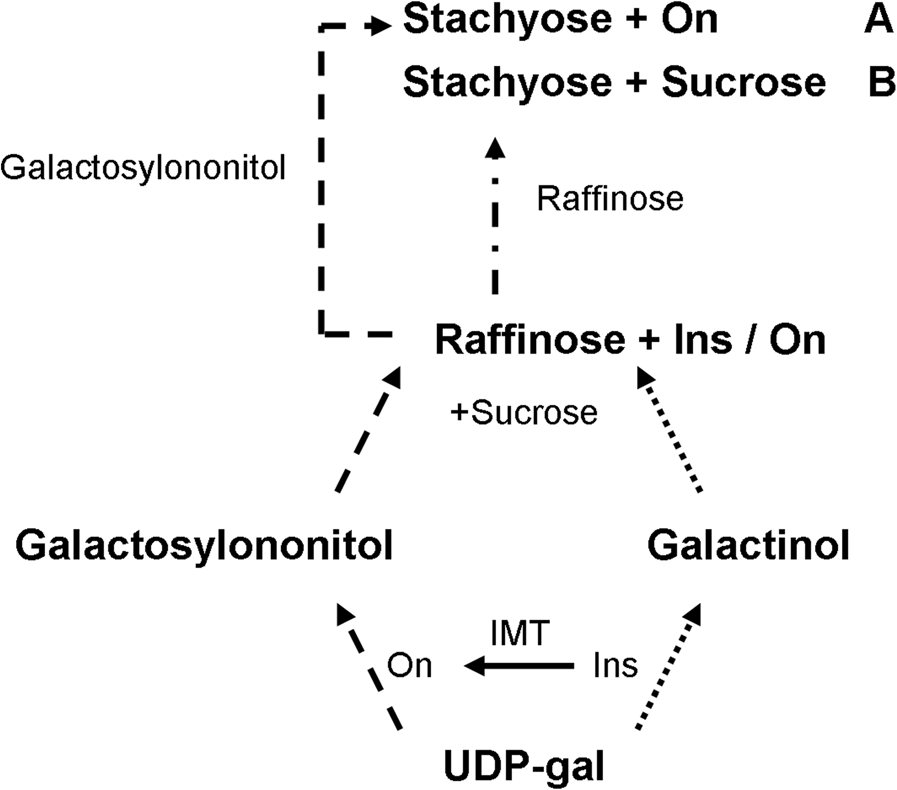
**Hypothetical model for the possible involvement of ononitol in the accumulation of stachyose and sucrose in *IMT *transgenic canola.** (**A**) Hypothetical production of stachyose and ononitol via galactosylononitol in *B. napus*; (**B**) Hypothetical production of stachyose and sucrose via raffinose:raffinose galactosyltransferase in *B. napus*. Dotted arrows indicate reduced biosynthesis. Dashed arrows indicate possible alternate routes. Dash-dotted arrows indicate probable direct galactosyl-residue transfer from raffinose. On, ononitol; Ins, *myo*-inositol; IMT, *myo*-inositol methyltransferase; UDP-gal, uridine diphosphate galactose.

In mature transgenic seeds, the level of Ins was not changed significantly (Figure 8) but presumably during maturation was partitioned among PhA, galactinol and ononitol biosyntheses (Figure 5B). Accordingly, PhA formation was decreased in concert with ononitol accumulation. This pathway perturbation (Scheme 1) would have lowered the production of galactinol as well, and consequently, RFO accumulation [40] with the notable increase in sucrose levels (Figure 8). Curiously, the decrease in raffinose levels was accompanied by an almost proportionate rise in stachyose. Since sucrose levels remained high in transgenic seeds, the inverse modulation in the levels of raffinose and stachyose could indicate raffinose as a possible galactosyl donor for the subsequent chain elongation in a raffinose:raffinose galactosyltransfer manner, in which a molecule of raffinose would release its sucrose moiety after each galactosyl-residue transfer to another raffinose molecule (Figure 9B) [41,42]. This is conceivable in view of the limited availability of galactinol (Figure 8). Alternatively, since methylated derivatives of galactinol are known to take part in RFO synthesis in some plants [43], and although not *hitherto* proven in *B. napus*, we propose that ononitol, the methylated product of IMT, may be utilized to form the corresponding galactosylononitol (methylated galactinol), which could still participate, in part, in RFO-chain elongation to the higher oligomer, stachyose (Figure 9A). Since this does not account for the observed increase in sucrose levels, we postulate that both routes may be working concertedly. The differential extent of stachyose and sucrose accumulation (Figure 8) could be indicative of the different kinetics of the two routes (Figure 9A and 9B).

## Conclusions

Early stages of *Brassica* seed development appear to be dominated by reciprocal interconversions of polar Ins phosphates and non-polar species. Although Ins is shown to be produced in significant amounts in the endosperm as well as seed coat during the early stages, no PhA accumulation occurs in those tissues. Instead, PhA accumulation appears to be mainly restricted to the embryo throughout seed development and maturation.

In addition to lowering PhA levels in the developing seeds, the competitive methylation of Ins resulted in changes in the distribution and accumulation patterns of seed carbohydrates, leading to enhancement of the digestible and metabolizable energy profile of the meal as demonstrated by the higher content of the nutritionally useful sugar, sucrose. While the ratio of raffinose to stachyose was altered, the overall balance of RFO appears to be unaffected. No deleterious effects were encountered as a result of Ins methylation in developing *Brassica* seed.

It is evident from this study as well as previous ones that one of the answers to reducing phytate in crop seeds to a level which allows an appreciable beneficial effect on phosphorous and microelements bioavailability without adversely affecting yield and phenotype may be found in a model which combines the additive effects of more than one mechanism acting concertedly with separate independent contributions. Such an approach would allow selective inhibition of Ins phosphorylation at different steps for optimum phytate reduction. Nonetheless, the current strategy, if adopted, could potentially raise the canola market value as well as that of other crops.

## Methods

### Plant material and chemicals

Ice plant (*Mesembryanthemum crystallinum*) seeds were germinated, grown, salt treated and sampled as described previously [20,44]. Seeds of *B. napus* (Westar) plants, grown under growth chamber conditions (16 hour day at 20°C/8 hour night at 15°C photoperiod) were harvested at maturation. Seeds at different developing stages were also collected. Developing seeds were separated into seed coats and embryos on ice under a binocular dissecting microscope. Fresh seeds were cut half open with a scalpel. Endosperm of 15 and 20 DAP was collected by pipette. The seed coat and embryos were washed with ultra pure water three times. The excess water was absorbed with filter paper. The developing seeds and dissected tissue were frozen immediately in liquid N_2_. These were freeze dried for 24 hours and then extracted for PhA and Ins analysis.

Ononitol standards were purchased from GlycoSyn Technologies, Lower Hutt, New Zealand.

### *In Vivo* labeling of developing seeds with ^*3*^*H-myo-*inositol

Siliques at different developmental stages (15–40 DAP) were cut from plants and the cut end immediately put into 10 ml sterile culture medium in 50 ml tubes supplemented with 5μCi ^3^H-*myo*-inositol and incubated in a growth chamber for two days. Seeds were harvested and crushed in liquid nitrogen then extracted consecutively with hexanes (lipid fraction), 0.5N HCl (phytate-containing acid-soluble fraction) and trifluoroacetic acid (TFA-soluble cell debris fraction). The radioactivity in each fraction was assessed in a scintillation counter.

### Phytic acid extraction

Mature canola seeds (350 mg) were homogenized in 4 ml of ammonia in methanol (10% w/w) [45], then vortexed. After a 10-minute incubation, 3 ml of hexanes was added and the samples were vortexed and centrifuged (RCF 2500). The liquid phases were discarded and the seed pellets re-extracted with 3 ml of hexanes, centrifuged and the supernatants discarded. Pellets were washed three times with 5 ml of absolute methanol, resuspended in 6 ml of 0.5 N HCl and kept at room temperature for 15 minutes. The slurry was centrifuged and the supernatant was filtered through a 0.45 μm GHP Acrodisk filter (Gelman Science) prior to HPLC analysis. At this stage samples were stored at -20°C. PhA was extracted from 50-100 mg of seeds at different developmental stages. For dissected material, seed coat (20 mg), endosperm (20 mg), embryo: 15 DAP (10 mg), 20 DAP (50 mg) and older (100 mg) was used. The extraction volume of HCl was adjusted proportionately.

### HPLC conditions for phytic acid analysis

Phytic acid analysis was performed on a Waters 660E multi-solvent delivery system equipped with in-line degasser AF, 717plus Autosampler and a Sedex 55 (S.E.D.E.R.E.) evaporative light scattering detector at 50°C, 2 bar, gain 7. The HPLC system was controlled and data processed by the Waters Millennium™ 2010 Chromatography Manager, version 2.15.01. Samples were chromatographed on an IC-Pak Anion HC, 150 mm × 4.6 mm column (WAT026770, Waters), at 22°C, with 100 mM nitric acid at a flow rate of 1.0 ml/min. A Waters IC-Pak Anion Guard-Pak (WAT010551) was used as the pre-column. A fritted filter guard (A-103X Rep Frit (BLK) 0.94 × 0.25 from Upchurch Scientific) was placed in front of the pre-column. Injections, typically 75 μl of undiluted sample, were in duplicate with a separate result generated for each injection. A calibration curve was prepared for each run with the levels of standards at 25, 50, 75 and 100 μg. Standards were prepared with phytic acid dodecasodium salt C_6_H_6_O_24_P_6_Na_12_·9H_2_O (Sigma), which was dissolved in the same concentration HCl as that used to extract the samples (0.5 N) to a concentration of 10 μg/μl. The concentrated solution was diluted to 1.0 μg/μl in 0.5 N HCl. Volumes of 25, 50, 75 and 100 μl of the diluted standard were injected in duplicate and phytic acid was detected at an average retention time of 3.9 minutes.

### HPLC conditions for carbohydrate analysis

Sugars were extracted from seeds, and analyzed by HPLC as described [40]. Briefly, duplicates of defatted tissue of ten seeds, unless otherwise indicated, were extracted with 80:20 v/v ethanol-water at 70°C for 30 min, followed by centrifugation and evaporation of the supernatant to dryness. Samples were reconstituted in 18-MΩwater, and filtered through 0.45 μm nylon filters prior to HPLC analysis. To accommodate analysis of early seed stages, extraction and reconstitution volumes were proportional to sample weight.

Galactinol, *myo*-inositol and D-ononitol were separated on a CarboPac™ MA1 column (4 mm × 250 mm) preceded by a CarboPac™ MA1 guard column (4 mm × 50 mm) with 500 mmol L^-1^ isocratic NaOH at 0.40 mL min^-1^ as eluent and detected by high performance anion exchange-pulsed amperometric detection (HPAE-PAD) using a Dionex ICS-3000 system (Dionex Corp., Sunnyvale, Calif.). Standards were galactinol, *myo*-inositol (Sigma-Aldrich, St. Louis, MO) and D-ononitol (GlycoSyn Technologies, Lower Hutt, New Zealand).

Glucose, galactose, fructose, sucrose, stachyose and raffinose were separated on a CarboPac™ PA1 column (2 mm × 250 mm) preceded by a CarboPac™ PA1 guard column (2 mm × 50 mm) and then an Amino-Trap™ guard column (2 mm × 50 mm) with 25 mmol L^-1^ isocratic NaOH at 0.25 mL min^-1^ as eluent and detected by HPAE-PAD. Standards were galactose, fructose, sucrose, raffinose, stachyose (Sigma-Aldrich, St. Louis, MO), and glucose (Fisher Scientific, Hampton, NH).

### Inorganic phosphate analysis

Inorganic phosphate (P_i_) in mature transgenic seeds was assayed using published protocols [46].

### Cloning of *IMT* and plant transformation

Fresh salt-stressed ice plant leaf tissue was frozen in liquid nitrogen and crushed to fine powder. Total RNA was extracted with TRIzol® Reagent (Invitrogen). Poly (A)^+^ RNA was isolated using published methods [47]. The first strand cDNA was generated using the Roche reverse transcription kit, and amplified with the sequence specific primers (forward, 5’-TTTTTGGATCCAAGAGAA AAAAAAATGACTACTTACACC-3' and reverse, 5’-TTTTTGCGGCCGCATAAAGGCAAATCATACACTG-3’) by PCR based on the published sequence (Accession No. M87340). The reaction was initiated by heating at 94°C for 2 min followed by 35 cycles of heating at 94°C for 1 min, annealing at 52°C for 1 min and extension for 3 min at 72°C. The PCR product (1418bp) was purified using a PCR purification kit (Promega) followed by digestion with *Bam*HI and *Not*I, whose sites were incorporated into the forward and reverse primers, respectively. The digested DNA fragment was subcloned into pSPORT1 (Invitrogen) and sequenced. The *IMT* gene was subcloned into pRD400 [48], which contains a napin promoter to produce plasmid pNIMT. Additional versions of the IMT gene, with modified translational context were produced [36,37] (Table 1). These as well as the parent-transgene were cloned into pRD 400 under the phaseolin promoter to generate pPhIMT1-3. All constructs were transferred into *Agrobacterium tumefaciens* strain GV3101 containing the helper plasmid pMP90 by triparental mating, followed by *Agrobacterium*-mediated transformation of *Brassica napus* (cv. Westar) [49].

### Over-expression of *IMT* in *E coli* and production of antibodies

The IMT cDNA fragment was cloned into the bacterial expression plasmid, pPROEXHTb (Invitrogen). Protein expression was induced by adding IPTG to the culture medium to a final concentration of 1 mM. The *E. coli* culture was harvested after a 3-hour incubation at 37°C. The His-tagged protein was purified with Ni-NTA Agarose (Qiagen) under denaturing conditions. The purified protein (43 Kda) was used to raise polyclonal antibodies against the IMT enzyme [50], which were subsequently used in Western analyses of transgenic lines.

### Southern and Northern blot analyses

Genomic DNA was extracted from leaf tissue using Wizard® Genomic DNA Purification Kit (Promega). RNA extraction from developing seeds of both transgenic and wild-type plants was conducted by using RNeasy plant total RNA kit (Qiagen). Southern and Northern analyses using Hybond-N^+^ membrane (Amersham) were performed essentially as described [51].

### IMT Enzyme assay and Western analysis in transgenic plants

Total protein extracts from developing seeds (approximately 40 DAP) as well as leaves of transgenic lines, and wild-type plants were assayed for IMT-enzyme activity exactly as described previously [20].

For Western analysis, total soluble proteins were extracted from developing seeds as described [44]. TND buffer, 90 mM Tris–HCl (pH 8.3 at 4°C), 9mM DTT and 2 mM Leupeptin (100 μl) was added to 50 mg of frozen seeds crushed in liquid nitrogen. Soluble protein samples were prepared by collecting the supernatants. Protein concentration in each sample was determined using the Bradford assay with BSA as the standard. Protein samples (15 μg each) were used in Western blot analysis. The samples were separated by PAGE and blotted on a Nitrocellulose membrane (Bio-Rad) [51]. The immune-reactions were conducted using Bio-Rad Immun-Blot® Assay Kit.

## Abbreviations

DAP: Days after pollination; Ptd-CMP: Phosphatidylcytosine monophosphate; PtdIns: Phosphatidylinositol; PI-K: Phosphatidylinositol kinase; PtdInsP: Phosphatidylinositol monophosphate; GolS: Galactinol synthase; RFO: Raffinose oligosaccharides.

## Competing interests

The authors declare that they have no competing interests.

## Authors’ contribution

FG, project PI, designed the concept, supervised all the experiments, contributed to and edited the manuscript. JD, WY, CB, KN performed the experiments and participated in the interpretation of results. WK supervised Brassica transformation experiments. All authors read and approved the final manuscript.
